# Effect of Temperature on Deformation and Fatigue Behaviour of A356–T7 Cast Aluminium Alloys Used in High Specific Power IC Engine Cylinder Heads

**DOI:** 10.3390/ma13051202

**Published:** 2020-03-07

**Authors:** Elanghovan Natesan, Stefan Eriksson, Johan Ahlström, Christer Persson

**Affiliations:** 1 Department of Industrial and Materials Science, Chalmers University of Technology, 412 96 Göteborg, Sweden; johan.ahlstrom@chalmers.se (J.A.); christer.persson@chalmers.se (C.P.); 2 Analysis and Verification, Volvo Car Corporation, 405 31 Göteborg, Sweden; stefan.a.eriksson@volvocars.com

**Keywords:** cylinder head, cast aluminium, mechanical properties, plasticity, fatigue, A356, deformation behaviour

## Abstract

Aggressive downsizing of the internal combustion engines used as part of electrified powertrains in recent years have resulted in increasing thermal loads on the cylinder heads and consequently, the susceptibility to premature thermo-mechanical fatigue failures. To enable a reliable computer aided engineering (CAE) prediction of the component lives, we need more reliable material deformation and fatigue performance data. Material for testing was extracted from the highly loaded valve bridge area of specially cast cylinder heads to study the monotonic and cyclic deformation behaviour of the A356–T7 + 0.5% Cu alloy at various temperatures. Monotonic tensile tests performed at different temperatures indicate decreasing strength from 211 MPa at room temperature to 73 MPa at 300 °C and a corresponding increase in ductility. Completely reversed, strain controlled, uniaxial fatigue tests were carried out at 150, 200 and 250 °C. A dilatometric study carried out to study the thermal expansion behaviour of the alloy in the temperature range 25–360 °C shows a thermal expansion coefficient of (25–30) × 10^−6^ °C^−1^. Under cyclic loading, increasing plastic strains are observed with increasing temperatures for similar load levels. The experimental data of the cyclic deformation behaviour are calibrated against a nonlinear combined kinematic–isotropic hardening model with both a linear and non-linear backstress.

## 1. Introduction

Aluminium–silicon (Al-Si) cast alloys are often used in the automotive industry due to their castability, high thermal conductivity and good mechanical and corrosion properties [1,2]. Alloying elements are added to tailor the alloy properties to better suit the application. Silicon is added in the alloy to improve the castability and flowability of the material besides improving the strength and ductility of the alloy [3]. Copper and magnesium addition in general tend to increase the strength while decreasing the ductility of the material [3]. A356 cast aluminium alloys are often used owing to their good castability, high strength and toughness while also having less susceptibility to casting defects in complex structures like cylinder heads [4,5]. A356 alloys with added Cu, like the material of interest in this study: A356 + 0.5% Cu, are used in structures like cylinder heads as they provide improved high temperature strength especially in the 200–250 °C temperature range owing to the precipitation of Al_2_Cu precipitates without significant reduction in ductility [6,7,8]. Other alloying elements are added in trace amounts to influence the shape, size and morphology of the secondary precipitates, to improve the strength and fatigue life of the material structure [9]. 

Besides the chemical alloying, various heat treatment procedures can also be used to significantly affect the mechanical and microstructural properties of the A356 alloy [10] and tailor the material behaviour to suit various application needs. Intermetallics of the alloying elements are often found as the concentrations exceed their solubility limit in the α–Al matrix. Such intermetallics can have various morphologies and formation times during solidification and the subsequent heat treatment and significantly influence the final material properties [3]. The A356 alloy is often employed in an overaged T7 [11] state to achieve a relatively stable microstructure and mechanical behaviour of the material [1,12]. The dendritic arm spacing primarily determined by the local cooling rate governs the ductility and strength of the material in the structure [13]. Since a complex structure like a cylinder head has varying solidification rates in different regions, the resultant microstructure and deformation behaviour have a high degree of variability within the structure [13,14].

Parts of the internal combustion engines (ICE) like cylinder heads are often subjected to cyclic thermo-mechanical loads during the engine start-stop cycles that induce severe plastic deformation because of the steep temperature gradients in the structure resulting in eventual fatigue failures. With the constant increase in the specific power of the internal combustion engines owing to engine downsizing, pressure charging and the increased number of start-stop cycles associated with the advent of the hybrid power trains, there is a greater need to address and computationally predict the failures associated with the thermo-mechanical fatigue loadings in the cylinder heads of the internal combustion engines [6]. To reduce testing and development costs, it is desirable to develop robust temperature dependent constitutive and fracture models to enable the design and fatigue life prediction of the structure through computer aided engineering (CAE) efforts. Cylinder heads typically experience temperatures up to 250 °C during operation [8,15,16] with different parts of the structure often at different temperatures and hence, it is critical to establish temperature dependent deformation and fracture models within the temperature range of interest [17]. To enable cost effective simulations, certain simplifications are often made, for example, previous studies have shown that the material of interest, cast aluminium A356, undergoes ageing at temperatures above 150 °C [12,17] and that fatigue lives could potentially be affected by the continued ageing of the material as shown by studies on aluminium alloys by Takahashi et al. [18] and Ovono et al. [19]. However, in place of the transient nature of the material behaviour, a shake-down of properties is assumed to simplify the simulation process and it has been shown to work without significant loss in accuracy while predicting the fatigue life of cylinder heads by research works on similar material [17]. The scatter in mechanical properties on account of the differing manufacturing parameters such as, cooling rates, mould differences among various others, is accounted for by establishing the deformation and fatigue behaviour of the alloy using samples extracted from multiple different cylinder heads and from regions sensitive to thermo-mechanical fatigue.

Since local yielding and significant plastic deformations are expected during thermo-mechanical fatigue (TMF) loading cycles, a strain-based approach to fatigue is often prescribed and is the approach adopted in the study. A strain based approach to fatigue life prediction in cases involving cyclic thermal stresses has been often found to be more suitable than other approaches to fatigue [20]. 

While a wide variety of chemical compositions of the A356 alloy are used and studied, there is dearth of relevant information on the deformation behaviour of A356 + 0.5% Cu–T7 group of alloys at high temperatures. Since these materials are often employed at such elevated temperatures, this study aims to establish the cyclic deformation and fatigue behaviour using completely reversed strain-controlled tests at total strain amplitudes of 0.2%, 0.3% and 0.4% at various temperatures. Further, tensile tests and dilatometric tests are performed at various temperatures to establish the monotonic deformation behaviour and the temperature dependent thermal expansion properties of the said alloy.

## 2. Materials and Methods 

### 2.1. Material

The material for testing was extracted from specially cast cylinder heads made of A356 + 0.5% copper cast aluminium alloys subjected to a T7 heat treatment. It is desirable to extract the test specimens for testing as close to the region of interest as possible to be able to examine the correct microstructure to accurately mimic the material deformation behaviour in the CAE simulations. The specimens used in this study were extracted directly from Volvo Cars’ (Gothenburg, Sweden) inline VEP4 series of engines with four cylinders. To ensure the high quality of the casts, the melt temperature was maintained quite low at temperatures between 690 and 710 °C with the die temperature maintained between 200 and 240 °C. The combustion chamber side was water cooled for quicker directional solidification and for obtaining a finer microstructure. The degassing procedure was carried out with graphite rotors and a steady flow of nitrogen gas at 2–10 litres per minute into the melt and rotor speeds of up to 300 rpm. The eutectic modification was controlled using strontium and the grain size was refined using titanium additions. The T7 heat-treatment involved solutionizing at temperatures of about 500–530 °C for about 3–5 h followed by quenching the structure in air. Further, artificial ageing was carried out at temperatures in the range of 200–230 °C for about 2–5 h.

**Figure 1:**
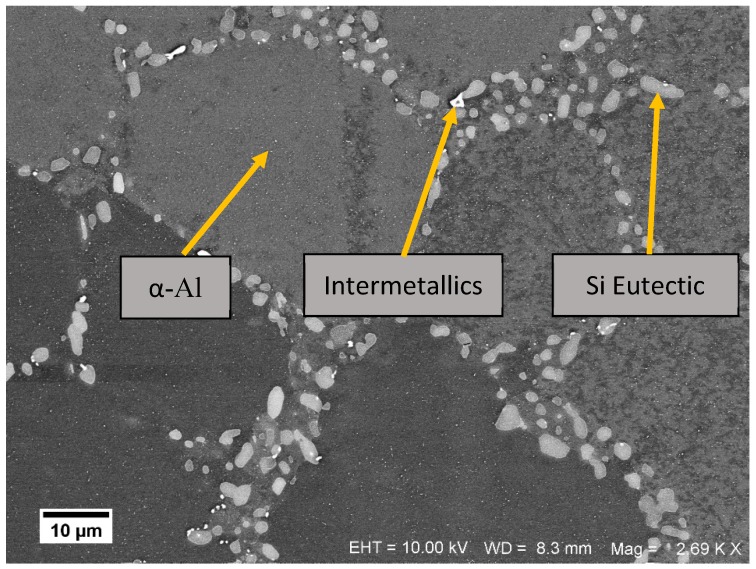
Scanning electron microscope image showing the multiple phases present in the A356–T7 + 0.5% Cu Alloy.

The average chemical composition of the alloy as determined using a modified ASTM E1251 [21] analysis of Al-base using optical emission spectrometry [22] on two specimens extracted from the cylinder heads used for testing is as presented in Table 1. The microstructure of the alloy indicating the primary α–aluminium phase forming the matrix, the Al-Si eutectic and the visible intermetallics of the Mg, Cu, Fe, Mn, etc., elements whose solubility exceed that of the solubility limit in the α–Al phase are shown in Figure 1. The material exhibits a dendritic microstructure with the primary aluminium skirted by the Al-Si eutectic punctuated with the said intermetallics with differing sizes and shapes in the different parts of the cylinder head. The secondary dendrite arm spacing determined using the mean linear intercept method on the aligned sets of the secondary cells was between 30 and 32 µm at the centre of the specimen and with about 5% decrease in the spacing closer to the mould walls where the associated cooling rates are higher. 

A phase analysis of the microstructure was carried out using a Zeiss LEO 1550 Scanning Electron Microscope (Zeiss, Jena, Germany) equipped with a Gemini field emission gun (Zeiss, Jena, Germany), a Centaurus back scattered electron detector (Thermo Fisher Scientific, Waltham, MA, USA) and a X-Max Energy-dispersive X-ray spectroscopy detector (Oxford Instruments, Abingdon, UK). The spherical particles observed in the eutectic region have silicon as the dominant constituent and the other identified phases are likely to be iron containing intermetallics given the inflated amount of iron content measured in them.

### 2.2. Sample Extraction

**Figure 2:**
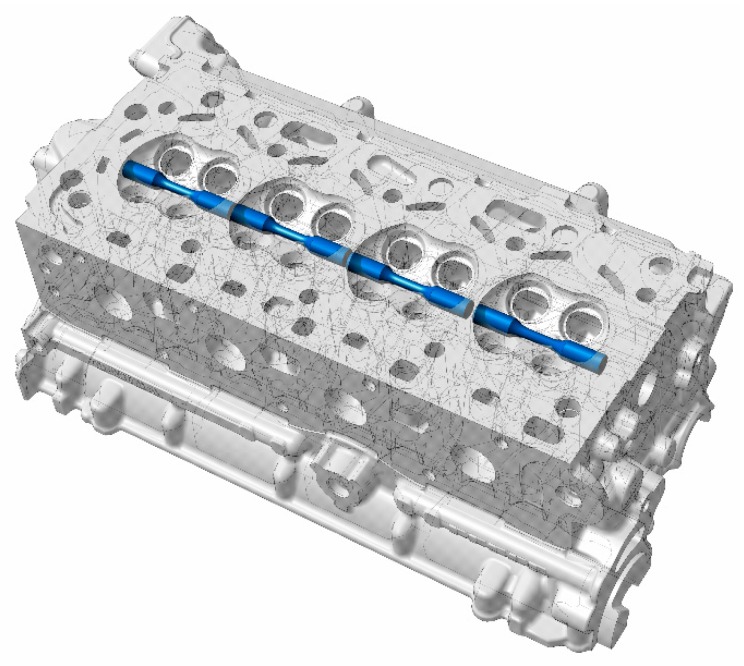
3D Computer aided design (CAD) rendering indicating the specimen extraction zones.

**Figure 3:**
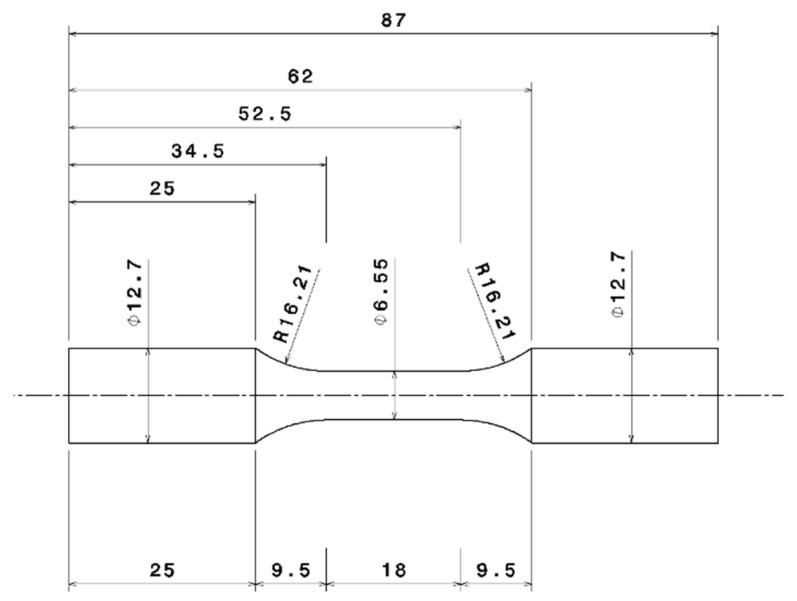
Test bar geometry (machined in accordance with ASTM standards).

Figure 2 shows the location of the extracted material that was used for all the testing in this study. The test bars were carefully extracted such that the test volume corresponded to the valve bridge area that is typically most sensitive to thermo-mechanical fatigue cracking in a cylinder head. The extracted material was machined according to the ASTM test standard recommendations to the geometry as shown in Figure 3. While it is possible to cast test specimens, it is often difficult to replicate the complex microstructural variations often found in the cylinder heads produced by industrial manufacturing processes [23] and hence the results from the samples extracted directly from the cylinder heads provide more representative test data for simulations.

### 2.3. Testing

#### 2.3.1. Test Equipment

An Instron 8501 servo hydraulic machine (Instron, Norwood, MA, USA) was used to perform the uniaxial tests to establish the monotonic and cyclic deformation and fracture behaviour of the alloy at various temperatures. The servo hydraulic machine was used in conjunction with a forced convection-based temperature chamber, Instron 3119–407 series (Instron, Norwood, MA, USA), for conducting the isothermal elevated temperature uniaxial tests. An Eurotherm 2408 controller (Eurotherm, Worthing, UK) was used with feedback from two K-type thermocouples in the temperature chamber to establish and self-regulate the target test temperatures. An external K-type thermocouple was used to measure the overall system accuracy placing it between ± 2.5 °C in the tested temperature range of room temperature (RT) to 300 °C. Either of two types of extensometers mounted on the gauge length were used for measuring the uniaxial strain. The tests at and below 150 °C were performed using the Instron 2620–603 axial clip-on dynamic extensometer (Instron, Norwood, MA, USA). The tests above 150 °C were performed using the Epsilon 3555–010M–020 high temperature axial capacitive extensometer (Epsilon Technology Corporation, Jackson, WY, USA). 

#### 2.3.2. Heat up and Temperature Stabilization

**Figure 4:**
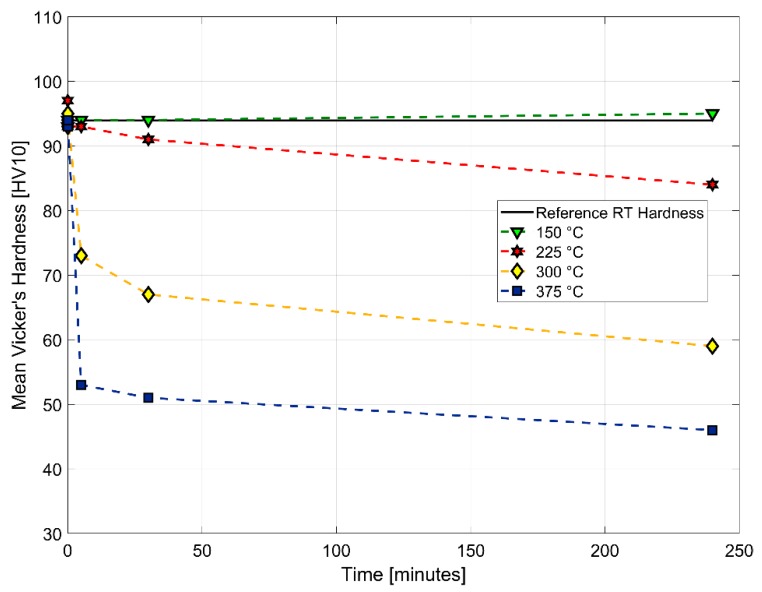
Ageing curves developed using Vickers hardness test (HV10).

There was significant thermal expansion of the equipment in the load string associated with the heating-up procedure. Before any high temperature experiments commence, it was imperative that the heat-up and stabilization procedures were followed rigorously to avoid interference from the equipment expansion on the measured sample deformation/strains during the tests. The temperature of the test chamber was increased to the target temperature with the specimen at a “no load” condition as the test equipment expanded gradually until the target temperature was reached and stabilization was achieved. A 3-hour period was used for the heat-up procedure for all temperatures and tests; it should be noted that the air temperature in the chamber was reached within a few minutes and the specimen followed quickly while the inherently larger mass grips and pull-rods required over an hour for stabilization. Internal studies [24] on the tested A356–T7 + 0.5% copper material ageing characteristics are summarized in Figure 4. The rate at which the tested material softened increased with increasing heat treatment temperatures and times at temperatures above 150 °C.

**Figure 5:**
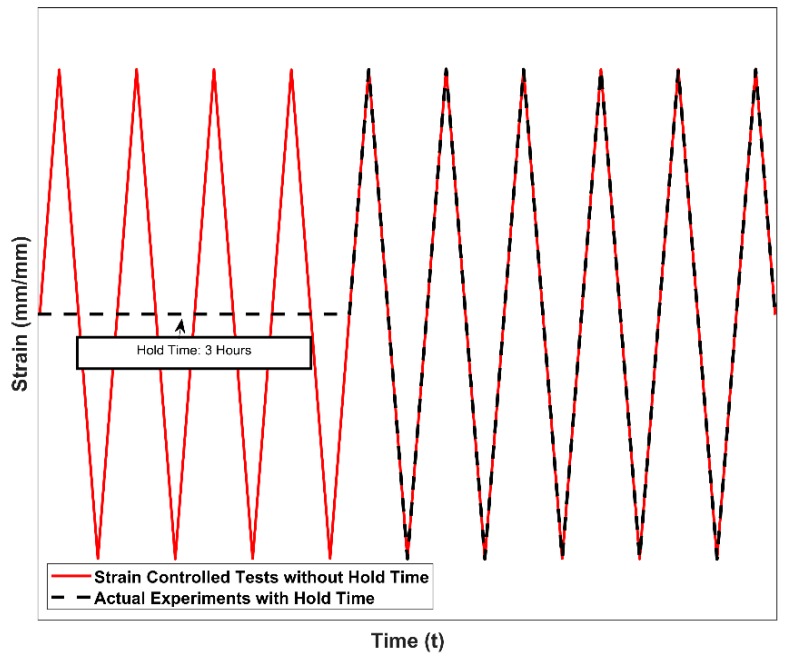
Load application in strain controlled low cycle fatigue (LCF) tests with and without hold time.

The rate of material softening increases with increasing temperatures and prolonged ageing times. With the above ageing results in mind, the hypothetical effects of the 3-hour hold time, used to achieve dimensional stabilization of the test set-up after the thermal expansion during the heat-up, can be schematically illustrated as shown in Figure 5. 

The samples subjected to the 3-hour hold time at the tested temperatures of 150, 200 and 250 °C are expected to have stable deformation behaviour during testing, i.e., the initial drop in the strength of the material is traversed and the stable plateau of the ageing curve is reached after the 180-minute hold time at the testing temperatures and this helps us isolate the cyclic ageing effects from the static ageing behaviour of the alloy to a reasonable degree.

#### 2.3.3. Sample Preparation and Testing

Tensile tests were performed in accordance with the ASTM E8/E8M tensile testing guidelines [25] using strain control at room temperature, 100, 150, 200, 250 and 300 °C with a minimum of 2–3 tests at each temperature with a prescribed strain rate of 0.01 %·s^−1^. The test samples were strained to fracture for all tests except at 300 °C to obtain the various monotonic tensile test data at the tested temperatures. The tests at 300 °C exceeded the extensometer tensile strain limit of 18% and hence could not be strained to fracture. The material stress response is presented till the threshold strain value tested. 

The strain controlled fatigue tests were performed in accordance with the ASTM E606/E606M standard [26] at three different temperatures of 150, 200 and 250 °C. The tests were carried out with three different total strain amplitude levels of 0.2%, 0.3% and 0.4% with a strain ratio of *R_ε_* = −1 and at a constant strain rate of 1 %·s^−1^. Each strain level at each of the temperatures is run with 2–3 replicas with a triangular load wave application to record the scatter in fatigue behaviour. To build the strain life fatigue curves at the different tested temperatures, all the tests were run to failure. A sample is considered to have failed when the peak stress amplitudes developed during a cycle had dropped below 75% of the peak stress amplitudes recorded in the 25th strain load cycle when sufficient “stabilization” in the peak stress values has been achieved. To minimize the effect of the surface finish of the tested samples on the measured fatigue lives, all the test specimens were polished sequentially using increasingly finer SiC papers. Fine polishing was carried out using a diamond suspension with 3 µm particles and oxide polishing was carried out using a colloidal silica suspension with 0.04 µm particles to obtain a mirror surface finish.

To perform thermo-mechanical CAE simulations, reliable thermal expansion data are required to be able to accurately model the thermal strains and stresses in the structure. To study the thermal expansion behaviour of the alloy, a dilatometric study was carried using NETZSCH DIL402C dilatometer (Erich NETZSCH GmbH and Co. Holding KG, Selb, Germany) employing a horizontal pushrod to determine the coefficient of thermal expansion of the material at different temperatures. The study was carried out using a cylindrical specimen with 10 mm diameter and 50 mm length with a smooth surface finish. The A356 + 0.5% Cu–T7 sample was subjected to a controlled temperature program wherein the temperature of the sample was raised from 25 °C to 360 °C with a heating rate of 1 °C min^−1^. The test was carried out in a nitrogen atmosphere and with a contact pressure of 0.3 N being exerted on the sample. A summary of all the tests included in this study is summarized in Table 2.

## 3. Results

### 3.1. Monotonic Deformation

**Figure 6:**
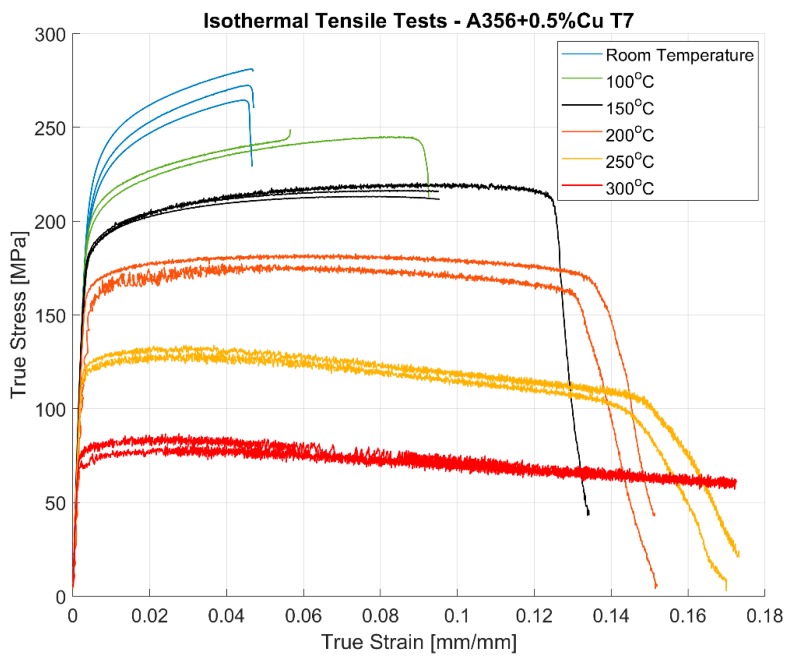
Tensile stress–strain curves at different temperatures of A356 + 0.5% Cu - T7 alloys.

The stress response of the strain controlled monotonic tests are presented in Figure 6. Since the deformation values are large, the true stresses and strains are preferred over engineering stresses and strains for the evaluation of the tensile curves. As with most metals, the tensile and yield strength of the material decreases with increase in temperature. On the other hand, the ductility of the material improves with increasing temperatures with the ductility measured at over 17% at 300 °C without tensile fracture. There is significant scatter in the stress–strain response between identical test replicas, often observed with cast structures with an inhomogeneous microstructure. It is also of interest to note that the scatter is much less pronounced at elevated temperatures compared to the room temperature tests.

The results of the tensile tests are summarized in Table 3. The stiffness, yield strength and the tensile strength all decrease with increasing temperatures with a rather sharp fall in properties at the highest tested temperature of 300 °C. Furthermore, of interest is the increasing strain at fracture with the ductility at 300 °C being almost four times than at room temperature. The changed behaviour is often related to enhanced dislocation creep, dislocation slide and annihilation and associated dynamic softening appearing at elevated temperatures [27], above 150 °C for the tested material.

### 3.2. Strain Controlled Fatigue Tests

#### 3.2.1. Stress Evolution

**Figure 7:**
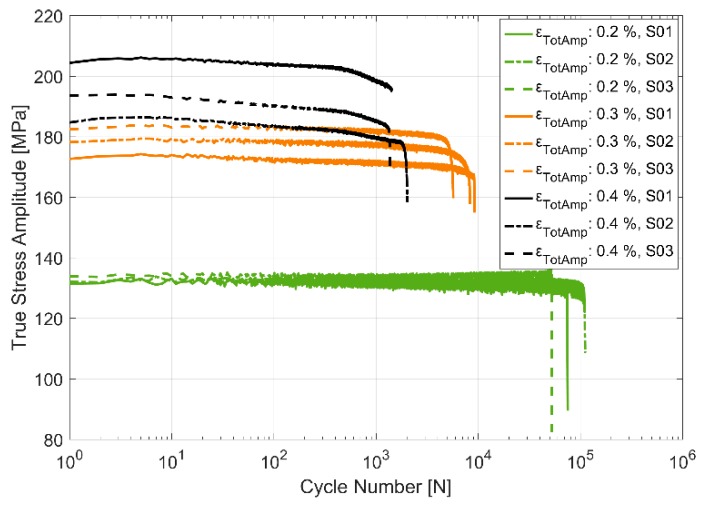
True stress amplitude evolution in strain controlled cyclic loading at 150 °C.

**Figure 8:**
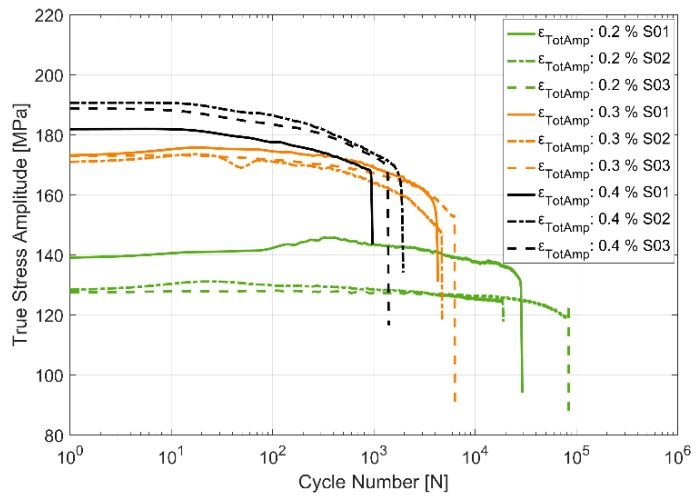
True stress amplitude evolution in strain controlled cyclic loading at 200 °C.

**Figure 9:**
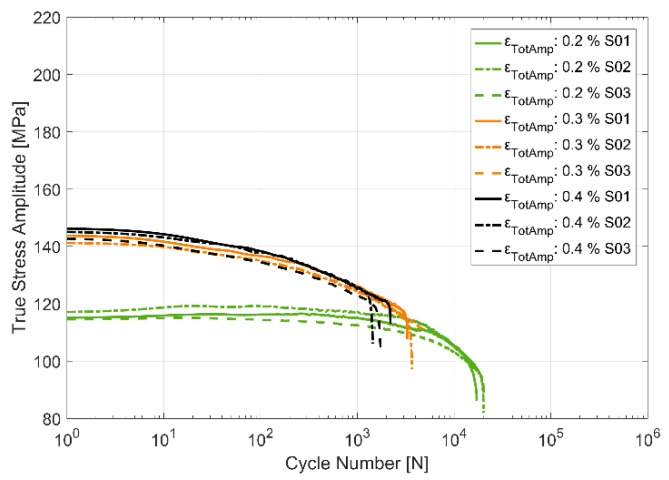
True stress amplitude evolution in strain controlled cyclic loading at 250 °C.

The stress response to the applied strain load cycles at the three different temperatures of 150, 200 and 250 °C and for the three different total strain load cycles of 0.2%, 0.3% and 0.4% are presented in Figure 7, Figure 8 and Figure 9. The material can be observed to soften cyclically with subsequent load cycles at all the presented temperatures with a higher degree of softening at higher temperatures. Furthermore, of interest to note is the decrease in scatter in the measured stress response and the life of the specimens between the test replicas at identical load cycles and temperatures with increasing temperatures. As can be expected, the flow stresses are reduced at higher temperatures than at lower temperatures for corresponding loads. As with most metals [20], the softening is more prominent in the initial stages of the cyclic loading, especially at higher strain loads.

#### 3.2.2. Hysteresis Loops

**Figure 10:**
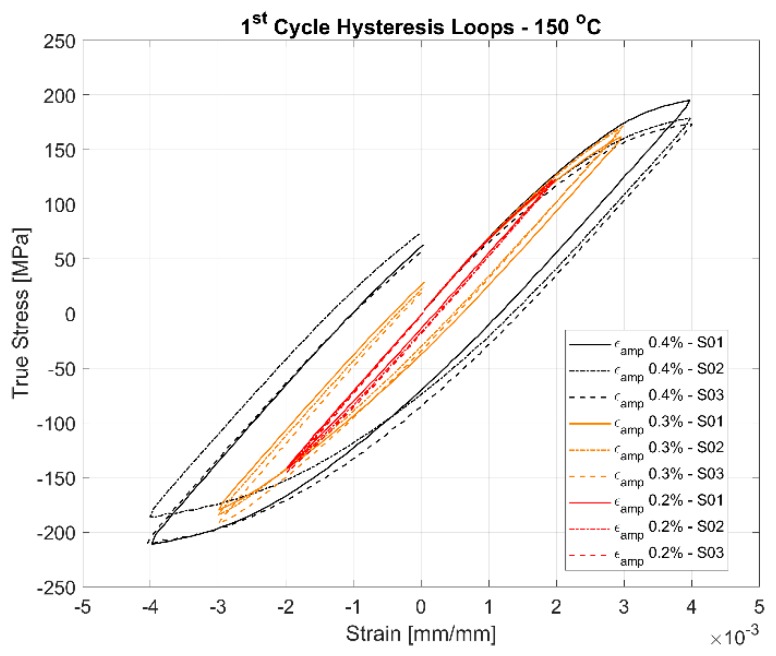
First cycle hysteresis loops of the strain controlled LCF tests at 150 °C with a strain ratio *R_ε_* = −1.

**Figure 11:**
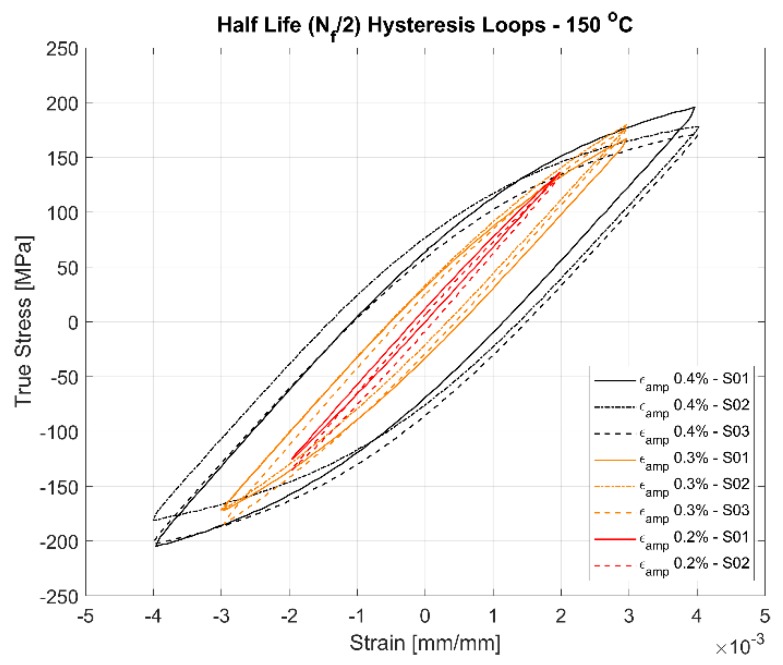
Half-life (N_f_/2) hysteresis loops of the strain controlled LCF tests at 150 °C with a strain ratio *R_ε_* = −1.

**Figure 12:**
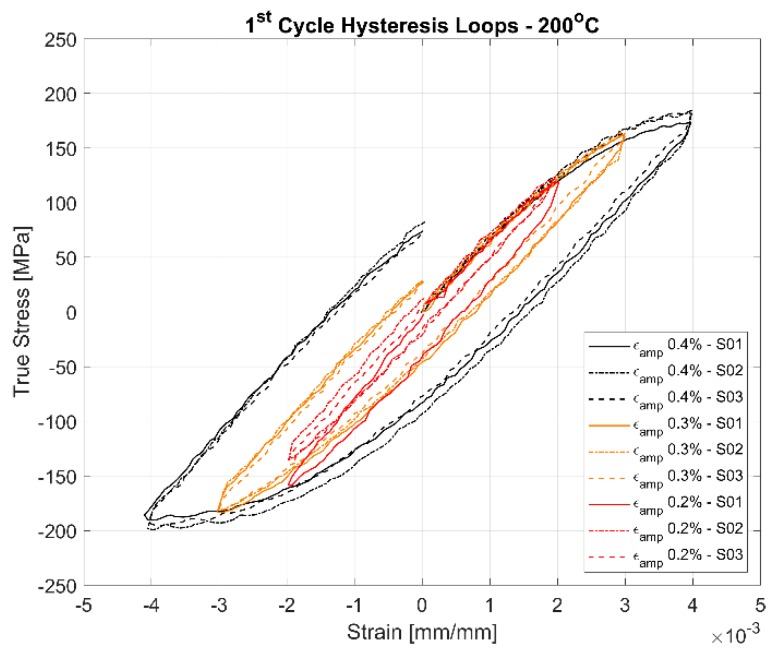
First cycle hysteresis loops of the strain controlled LCF tests at 200 °C with a strain ratio *R_ε_* = −1.

**Figure 13:**
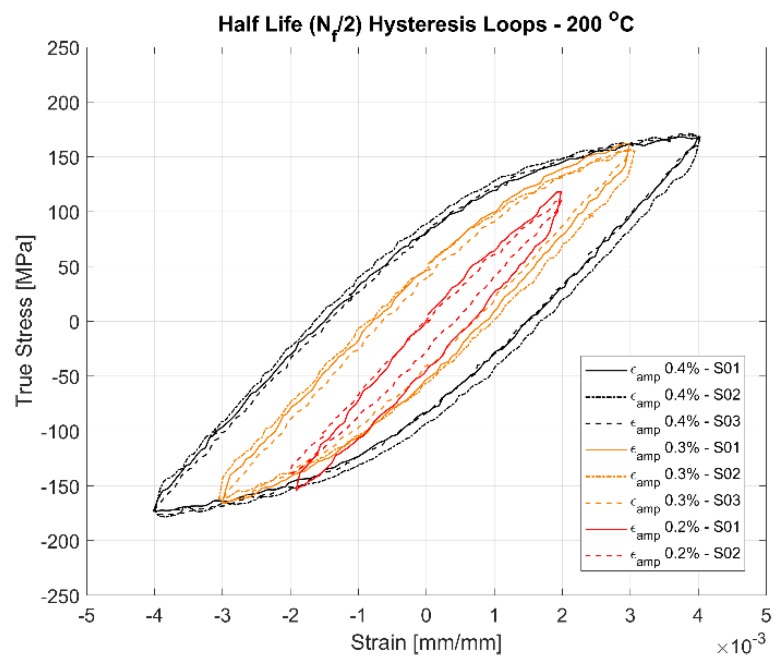
Half-life (N_f_/2) hysteresis loops of the strain controlled LCF tests at 200 °C with a strain ratio *R_ε_* = −1.

**Figure 14:**
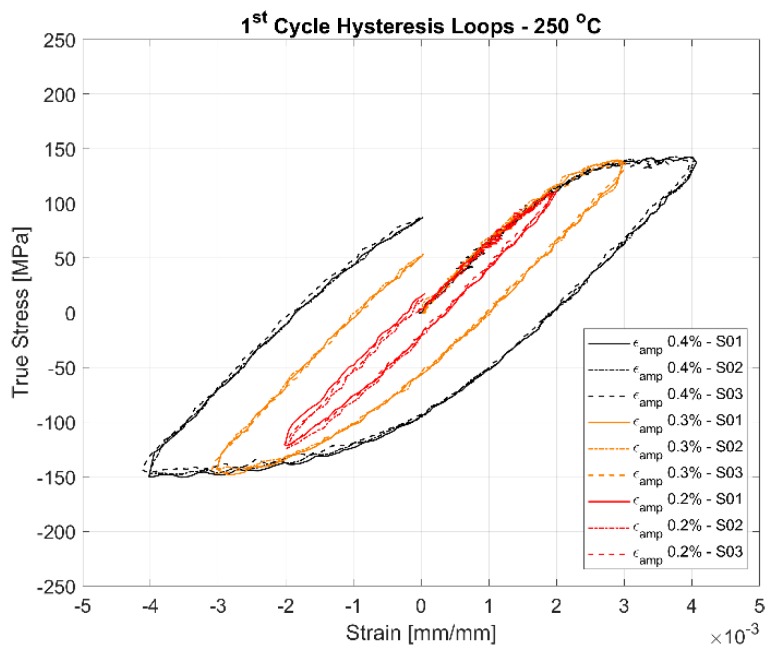
First cycle hysteresis loops of the strain controlled LCF tests at 250 °C with a strain ratio *R_ε_* = −1.

**Figure 15:**
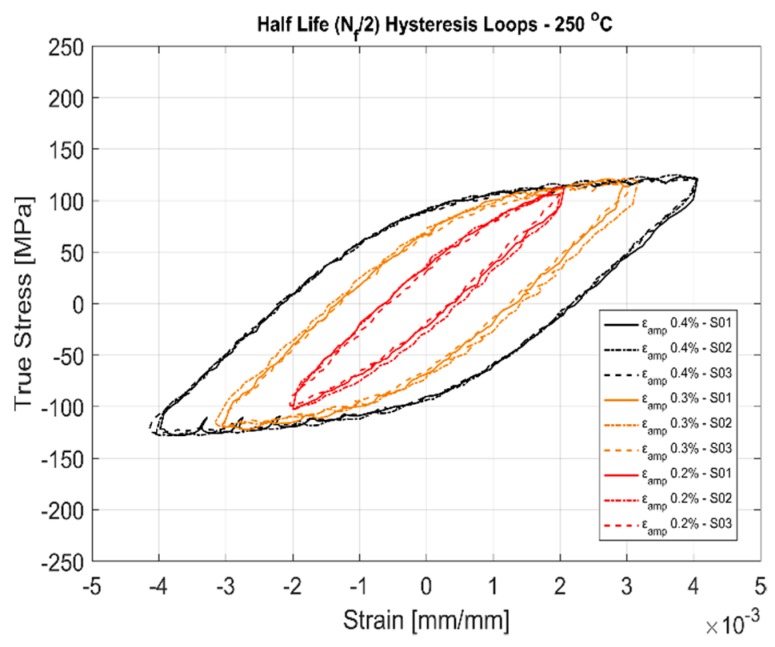
Half-life (N_f_/2) hysteresis loops of the strain controlled LCF tests at 250 °C with a strain ratio *R_ε_* = −1.

The first cycle and the stable half-life hysteresis loops presented in Figure 10, Figure 11, Figure 12, Figure 13, Figure 14 and Figure 15 respectively show the cyclic deformation for the various strain-controlled tests at the three different loads and temperatures. The hardening part of the hysteresis loops at isothermal temperatures show slight variations in their slopes at lower temperatures between different load levels and between replicas at identical loads. Like with most of the deformation properties presented before, the difference in the deformation behaviour between the replicas at different load levels decreases with increasing temperatures indicating the increased influence of the viscous deformation behaviour that overshadows the plastic deformation properties with increasing temperatures. An increasingly softening response is exhibited by the material with increasing temperatures as indicated by the decreasing peak stresses and the increase of the plastic strain amplitude indicated by the width of the hysteresis loops.

#### 3.2.3. Effect of Temperature on the Hysteresis Loops

**Figure 16:**
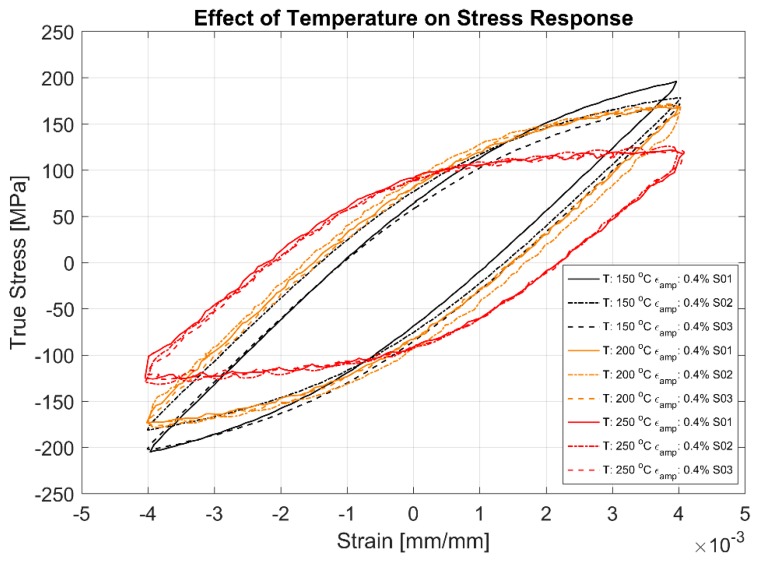
Half-life (Nf/2) hysteresis loops of the strain controlled LCF tests at 150, 200 and 250 °C with a strain ratio *R_ε_* = −1.

Figure 16 shows the effect of temperature on the deformation characteristics of the alloy at various temperatures. It is evident that the material softens with increasing temperatures and the plastic flow associated hardening also exhibiting lower stress response at higher temperatures. The softening with temperature is further evidenced by the plastic strain amplitudes, indicated by the width of the hysteresis loops being significantly higher at higher temperatures.

#### 3.2.4. Plastic Strain Evolution

**Figure 17:**
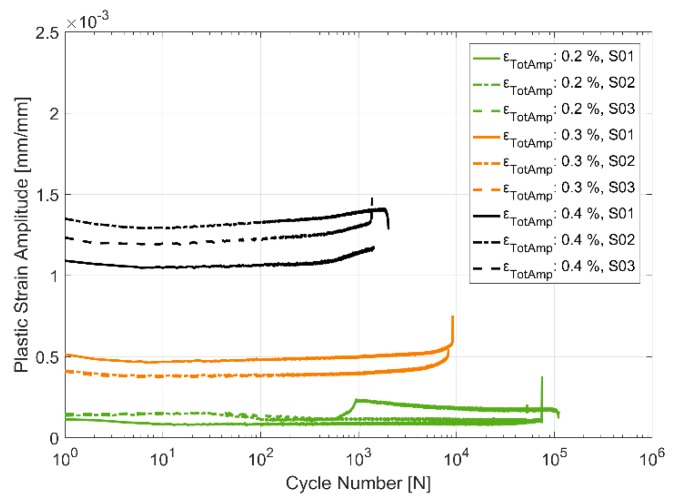
Plastic strain amplitude evolution in strain controlled cyclic loading at 150 °C.

**Figure 18:**
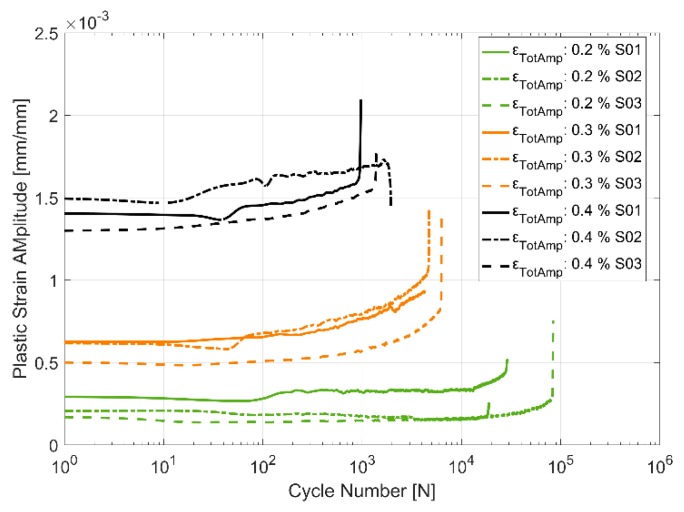
Plastic strain amplitude evolution in strain controlled cyclic loading at 200 °C.

**Figure 19:**
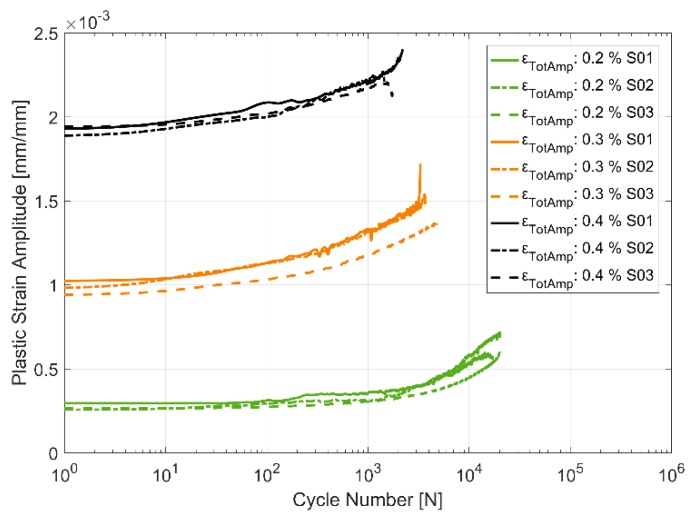
Plastic strain amplitude evolution in strain controlled cyclic loading at 250 °C.

An alternative way to look at the material hardening/softening behaviour is to observe the evolution of the plastic deformation with cyclic loading. The plastic strain amplitude is taken to be half the width of the hysteresis loop about the zero-load axis. Figure 17, Figure 18 and Figure 19 present the obtained plastic strain amplitudes against the applied strain load cycles. Just like the stress response in monotonic and cyclic loading, the plastic strain amplitude evolution exhibits a lower level of scatter between identical replicas at higher temperatures. While the cyclic softening, indicated by the increase in the developed plastic strain amplitude, is almost negligible at 150 °C, the notion that material softening increases with increase in temperatures is further evidenced by the evolution of the plastic strain amplitude at 200 and 250 °C. The applied load levels do not seem to have a significant effect on the rate of softening with all three load levels indicating similar trends in softening through the life of the component. The material exhibits insignificant plasticity at the lowest strain load level of 0.2% and at lower temperatures and increases significantly to contribute more than half the applied strain at the maximum total strain amplitude of 0.4% at 250 °C. Furthermore, of note is the noise/perturbations at certain points in the measured plastic strain amplitudes in the presented curves which could possibly be attributed to slightly varying temperatures of the extensometer during the high temperature tests as it is the most sensitive experimental measurement apparatus.

#### 3.2.5. Cyclic Yield Evolution

**Figure 20:**
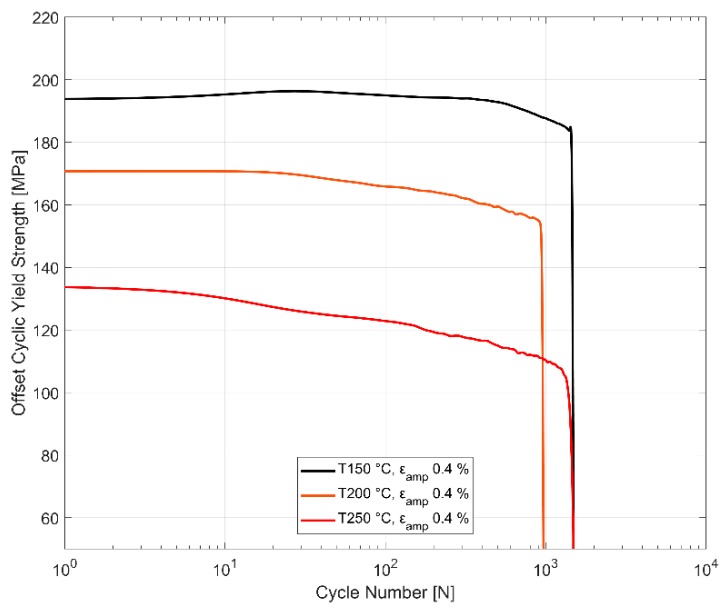
Influence of temperature on cyclic tensile yield strength evolution at ε_amp_ 0.4%.

To study the change in the size of the yield surface with cyclic loading, i.e., the isotropic hardening, it is of interest to study the evolution of the yield strength with subsequent load cycles. Figure 20 presents the evolution of the offset yield strength about the neutral load axis under the tensile loading part of the strain load cycles. At 150 °C, the material hardens slightly in the initial few cycles before softening through the subsequent load cycles until failure. At the other two elevated temperatures, the material exhibits softening behaviour from the start of the cyclic loading. Furthermore, as with all the previously studied mechanical properties, the evolution of the cyclic yield strength also exhibits significantly less scatter between identical replicas at elevated temperatures. The softening rate at the elevated temperatures show a non-linear behaviour with steeper softening rates in the initial part of the life compared to the latter portion where the softening seems to stabilize to a constant rate.

### 3.3. Dilatometry-Coefficient of Thermal Expansion vs. Temperature

**Figure 21:**
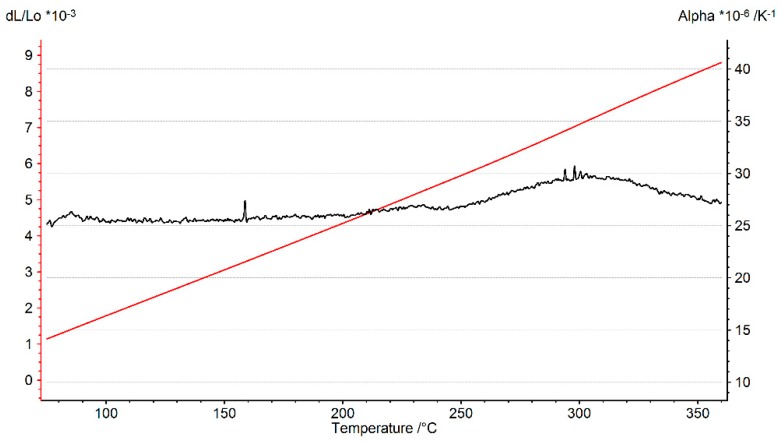
Dilatometry curve presenting the thermal strain and instantaneous coefficient of thermal expansion against temperature.

**Figure 22:**
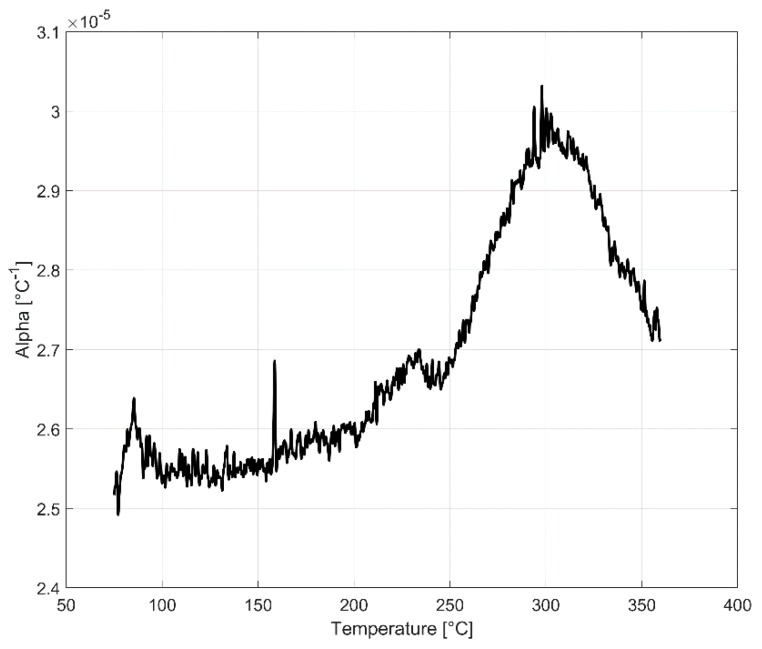
The variation of coefficient of thermal expansion with temperature.

**Figure 23:**
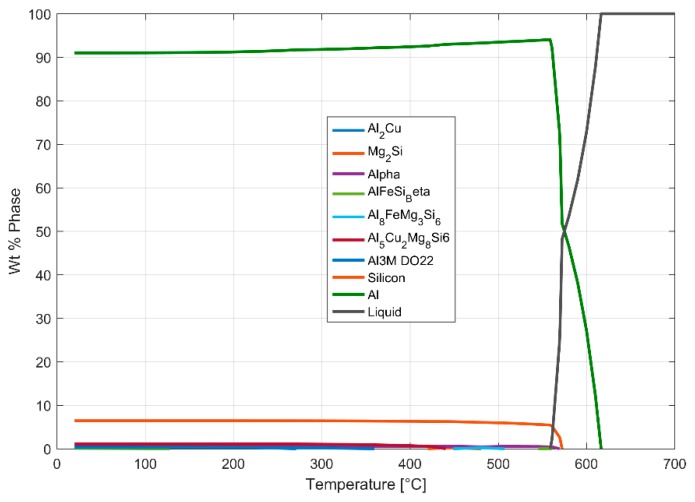
JMatPro simulation showing the mass fraction of the phases expected at various temperatures for a nominal chemical composition of Al-6.8Si-0.35Mg-0.1Fe-0.12Ti-0.07Mn-0.53Cu.

**Figure 24:**
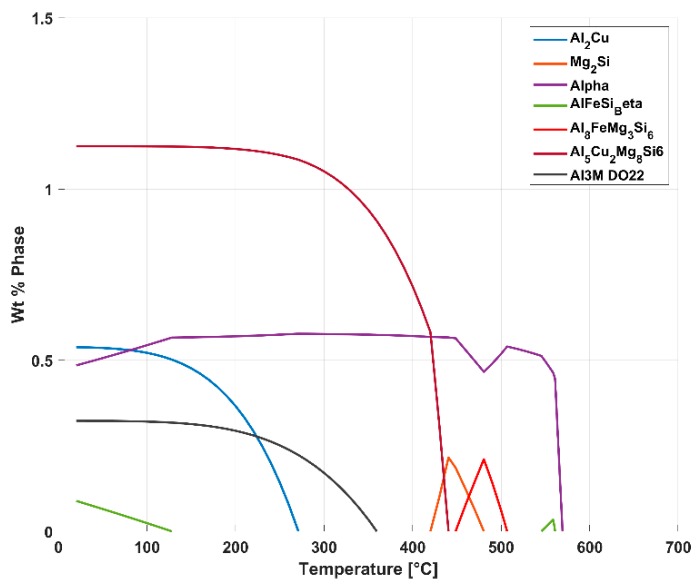
Magnified image showing the presence of low volume intermetallic compounds in the alloy.

The results of the dilatometry test to study the variation of the coefficient of thermal expansion with temperature is presented in Figure 21 and Figure 22. The thermal expansion remains fairly stable through the temperature range with the coefficient of thermal expansion varying in between (25–26) × 10^−6^ °C^−1^ in the temperature range between 25 °C and 250 °C. The linear thermal expansion coefficient increases rapidly up to 30 × 10^−6^ °C^−1^ as the temperature increases further from 250 °C to 300 °C before receding with further increase in temperature. From Figure 4, we could see that the ageing kinetics is higher at temperature above 250 °C and that accelerated phase transitions could perhaps explain the change in the measured coefficient of thermal expansion at such elevated temperatures. The equilibrium phase diagram indicating the meta-stable equilibrium phases expected through thermodynamic simulations using the JMatPro software (Sente Software Ltd., Surrey, UK) is as shown in Figure 23 and Figure 24. Aluminium (≈90%–93%) and silicon (≈6%–7%) are the dominant phases while the intermetallics form the rest. The simulation also shows the expected presence of Al_2_Cu phase to which the higher elevated temperature strength of the A356–T7 + 0.5% Cu is often attributed to. The measured thermal expansion values for the A356 + 0.5% Cu–T7 alloy seems marginally higher than those reported by Grieb et al. [28] for similar group of alloys.

## 4. Discussion

### 4.1. General Discussion

The dendritic arm spacing, size, shape and morphology of the secondary phases are a function of the chemical composition, cooling rates and the heat treatment and it tends to vary over the geometry of the cylinder head as shown in Figure 2. All the micrographs and other measurements presented in this work pertain to the highly loaded valve bridge area of the cylinder head. A cylinder head has a highly complex geometry with the material thickness differing from region to region. When such an intricate structure is cast, the cooling rate is different in different parts of the mould. As a result, the dendritic arm spacing and consequently, the deformation behaviour, damage mechanisms and the fatigue lives of the material varies through the geometry [1,3] and is highly dependent upon where the test specimens are extracted from. It is of interest to study the deformation behaviour of the material that is critically loaded in the thermo-mechanical fatigue (TMF) context that which is the focus of the current research work. Numerous studies have shown that the material in the valve bridge areas of the cylinder head experience the steepest thermal gradients during the engine start–stop cycle and tends to crack first [4,15,16] and hence is the region of interest in this study.

Studies by Tabibian et al. [12] on A356 + 0.5% Cu–T7 alloys have shown material over-ageing at temperatures above 150 °C similar to results obtained from studies at the author’s lab [24]. It is, hence, critical to keep track of the over-ageing for 3 h the test specimens were subjected to during the heating and equipment stabilization stage before the commencement of the tests. While all the results at 150 °C are not expected to differ significantly from ideal tests without pre-ageing, the mechanical response obtained and presented for temperatures above 150 °C are expected to be influenced by the pre-ageing to varying degrees depending on the temperature of the test given that the hold time of 3 h was identical for all tests. 

### 4.2. Monotonic Deformation

The deformation behaviour in Al–Si cast alloys is often rationalized by considering them akin to metal matrix composites [29] with α–Al acting analogous to the matrix and the eutectic Si, Mg, Cu, Mn, Fe intermetallics acting like the reinforcing phase [3]. During the deformation, as the particulate intermetallics remain elastic, a stress incompatibility is generated between the intermetallics and the soft matrix. A plastic relaxation occurs near the tip of the intermetallics with further deformation. At lower temperatures, it is reasonable to assume that the load shedding to the precipitates is higher and the intermetallics crack earlier before the commencement of plastic relaxation compared to corresponding strains at elevated temperatures thus reducing the ductility of the material with decreasing temperatures.

The ductility of the cast aluminium alloys is also reduced by the size and morphology of the precipitates with large and long intermetallics having a deleterious effect on the ductility [3]. Slower cooling rates promote the growth of the intermetallic precipitates [30] during solidification and owing to the difficulty in breaking up such large precipitates during the heat treatment cycle [31], they tend to reduce the ductility of the material. Since the extracted samples have slightly differing microstructures depending on the location of extraction from the cylinder heads, the deformation and ductility characteristics tend to vary between test replicas as evidenced by the monotonic deformation curves especially at lower temperatures as presented in Figure 6. This difference in ductility and deformation behaviour, however, seems to decrease between identical tests as the temperature is increased.

The effect of temperature on the measured strength and ductility of the material can be summarized to decreasing strength and increasing ductility with increasing temperatures. New deformation mechanisms and multiplication of the available slip systems at elevated temperatures contribute to the increased ductility with increasing temperatures [27,32,33]. The reduction of the peak stresses developed at elevated temperatures is also attributed to the enhanced mobility of the dislocations and their annihilation at increasing temperatures. These phenomena promote dynamic softening and consequently reduce the peak stress response of the material at higher temperatures [27,34].

Studies by Zhu et al. [2] of the A356–T6 alloy without significant copper additions shows expected reduced strength and enhanced ductility at room temperature in comparison to the tested alloy of A356 + 0.5% Cu–T7. Azadi et al. [35] studied the monotonic deformation properties of A356–T6 peak aged alloys at different temperatures and reports yield strengths and tensile strengths that are considerably higher even at elevated temperatures of 250 °C with lower copper addition in the alloy (0.01%) as compared to the tested A356 + 0.5% Cu–T7 alloy in this study. However, the ductility of the peak aged A356–T6 alloy was not presented by the author and hence it is impossible to compare the effect of copper addition and the state of ageing (T6/T7) on the ductility of the final material.

The scatter in the deformation behaviour between replicas can possibly be attributed to the difference in the microstructural variation between the samples, the inter-dendritic arm spacing in particular, which varies significantly even within the cylinder head structure. Numerous studies have shown the effect of the variation of the dendritic arm spacing on the resulting deformation properties [2,14,36,37,38].

### 4.3. Cyclic Deformation

**Figure 25:**
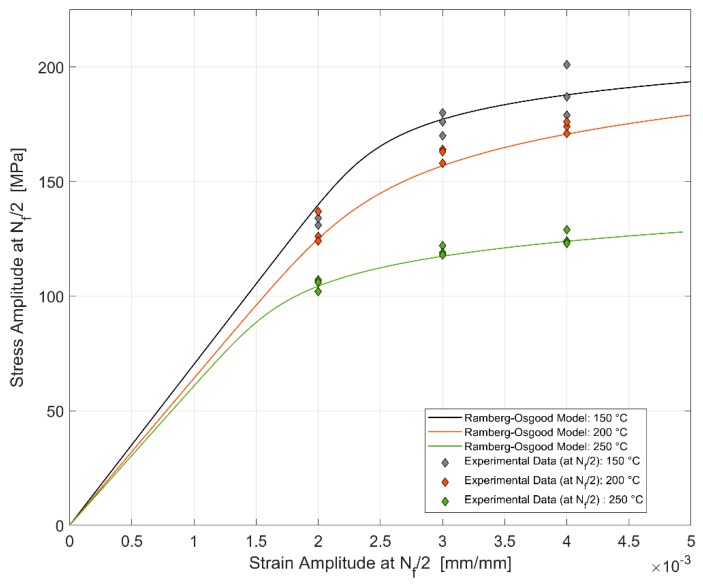
Effect of temperature on the cyclic stress–strain curve.

The cyclic hardening curve is usually used to represent the stable stress response to cyclic strain loads and vice versa. With the load and response levels being sufficiently symmetric, the corresponding amplitudes at half-life (N_f_/2) have been used to plot the so-called cyclic stress–strain curves. The data obtained from the half-life hysteresis loops of the different strain controlled LCF tests enable us to plot the cyclic stress–strain curves for the different temperatures and are presented in Figure 25. Since the cyclic hardening curves usually deviate smoothly from linearity [20], the Ramberg–Osgood type model has been used to describe the cyclic stress–strain curves.

Ramberg–Osgood Model for cyclic hardening:

(1) εa=σaE+(σaH′)1n′

where the constant *H′* and the cyclic strain hardening exponent *n′* are obtained by fitting the obtained data to the expression as shown below:

(2) $$\sigma_{a}=H^{\prime }\varepsilon_{pa}^{n^{\prime }}$$

The offset yield strength σ_₀_′ for the cyclic stress–strain curve is obtained by substituting a plastic strain amplitude of 0.002 in the expression obtained above. The obtained values for the cyclic stress–strain curves are presented in Table 4. Alloy processing has been shown to have a profound effect on the cyclic stress–strain behaviour. Metals that have been softened by heat treatment, like the over aged T7 temperature in the present case, and precipitation hardened aluminium alloys in general, often tend to harden with increasing strain amplitudes when cyclically loaded.

**Figure 26:**
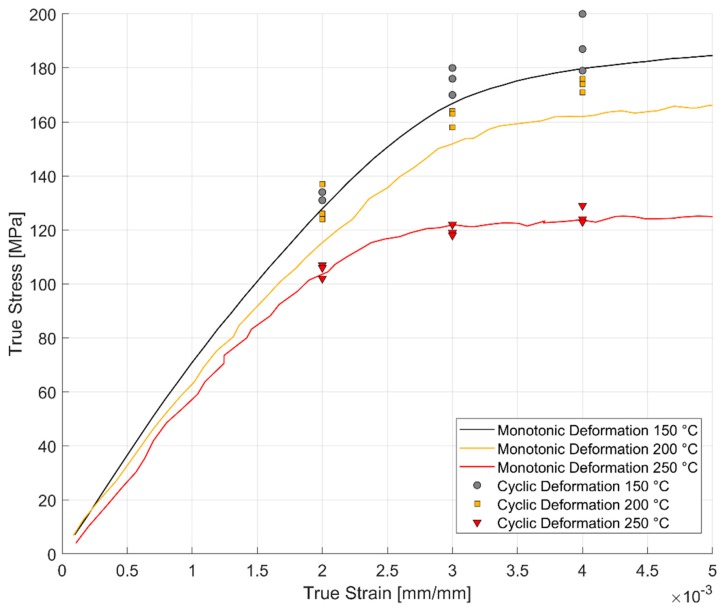
Comparison of the monotonic vs. cyclic stress–strain curve at elevated temperatures.

The comparison between the monotonic and cyclic hardening curves is presented in Figure 26 below. The nature of loading, monotonic or cyclic, seems to have an effect on the stress response at lower temperatures than at the higher temperature of 250 °C. The dislocation cell structures developed during monotonic and cyclic loading have been observed to influence the stress response of the material at lower temperatures by Snowden [39] and Grosskreutz [40]. At the higher temperatures, the creation and annihilation of the dislocations are enhanced and the associated dynamic softening is higher [27] which could explain the reduced work hardening with both the monotonic and cyclic loads. 

#### 4.3.1. Effect of Strain Amplitudes on the Hysteresis Loops

A comparison of the first load cycle presented in Figure 10, Figure 12 and Figure 14 and the hysteresis loops at half the life presented Figure 11, Figure 13 and Figure 15 show the hardening behaviour evolution of the A356 + 0.5% Cu–T7 alloys at different temperatures. At 150 °C, the stress response and the plastic strain amplitudes exhibit a trivial difference indicating insignificant cyclic hardening or softening response. At further high temperatures of 200 °C and 250 °C, the material exhibits significant softening with the plastic strain range increasing in width with increasing temperatures further indicating a temperature dependent softening. However, the strain load level dependence of the hardening slopes and the deformation difference between replicas tend to decrease with increasing temperatures.

Also, of interest is the differing hardening slopes between the strain levels at half-life compared to the first cycle hysteresis loops where the initial deformation slopes are similar. From Figure 10, Figure 11, Figure 12, Figure 13, Figure 14 and Figure 15, we can observe that a strain load level dependent hardening exists for temperatures up to 200 °C indicated by the differing deformation slopes of the half-life hysteresis loops compared to the corresponding first cycles. At the highest tested temperature of 250 °C, all the strain load levels exhibit similar hardening behaviour. This load dependent hardening behaviour at temperatures below 250 °C shows similarities to studies by Snowden [39] and Grosskreutz [40]. Snowden [39] studied the dislocation arrangements in aluminium crystals during cyclic hardening and observed that the ratio of the Bauschinger strain to the total applied strain amplitude increased with decreasing strain amplitudes. This higher reversibility of dislocations (Bauschinger effect) at small strain amplitudes, he contends, is a probable explanation for the marked difference in the hardening behaviour at differing strain amplitudes. Grosskreutz [40] also observes a differing dislocation structure depending on the applied strain levels. At low strain levels, during the hardening stage, the density of the dislocation bundles was found to increase while the spacing between them decreased and with most of the dislocations belonging to the primary system. At higher applied strain amplitudes, the dislocations within these bundles were fragmented to shorter lengths with the density of dislocations in the secondary slip system being increased and a rough three-dimensional structure was built up. The non-linear nature of hardening is explained by the negligible concentration of the point defects and clusters in the initial cycles that become significant at later stages and influence the flow stress by exerting increased frictional drag on the dislocation motion.

#### 4.3.2. Constitutive Modelling of Cyclic Deformation

To perform structural analysis of the cylinder heads, we need a constitutive model that relates the state of stresses and strains. By coupling the boundary conditions, loads and the material model, we can determine the mechanical response and life of the structure. The complexity of the model is determined by the degree of accuracy expected while keeping the computational costs low. Some of the factors affecting the constitutive model being the nature of the material, temperature, nature of the loading, etc. [41]. 

##### Cyclic Plasticity: Nonlinear Combined Isotropic–Kinematic Hardening Model

Figure 10, Figure 11, Figure 12, Figure 13, Figure 14 and Figure 15 indicate that the stresses are asymmetric about the null load axis with stresses in compression slightly higher than in tension at the corresponding peak strain loads. This indicates the need for a kinematic hardening model that takes in to account the translation of the yield surface when the load directions are changed. Figure 20 shows the evolution of the yield strength with successive load cycles indicating a continuous change in the size of the yield surface till fatigue failure of the material. Such a notion of a continuously changing yield surface size is also reinforced by observing the change in the width of the hysteresis loop, i.e., the plastic strain amplitude, for the different strain load cycles as indicated by Figure 17, Figure 18 and Figure 19. This necessitates an isotropic hardening model to be combined with the kinematic hardening model. The figures also exhibit differing hardening rates with the hardening in the initial cycles eventually decreasing to a saturation value indicating that a non-linear combined isotropic and kinematic hardening is quite suitable to model the cyclic deformation behaviour of the tested A356 + 0.5% Cu–T7 material.

A rate independent non-linear combined isotropic - kinematic hardening model as implemented in a commercial FE software like Abaqus [42] is used in this study to identify the model parameters. The model consists of a non-linear kinematic hardening term α and an isotropic hardening component.

##### Non-Linear Kinematic Hardening Model

The kinematic component consists of a pure kinematic term based off the linear Ziegler hardening law and a recall term that introduces the non-linearity. The model implemented has two kinematic backstresses that are superposed (one linear and one non-linear term) to get better predictions. The linear Ziegler hardening law with multiple backstresses is defined as below [42]:

Kinematic Hardening Law:

(3) $${\dot{\alpha }}_{k}=C_{k}\frac{1}{\sigma^{0}}\left(\sigma -\alpha \right){\dot{\bar{\varepsilon }}}^{pl}-\gamma_{k}\alpha_{k}{\dot{\bar{\varepsilon }}}^{pl}$$

Overall backstress:

(4) $$\alpha =\overset{2}{\underset{k=1}{\sum }}\alpha_{k}$$

where C is the initial kinematic hardening moduli and γ determines the rate at which the kinematic hardening moduli decreases with increasing plastic deformation. 

##### Non-Linear Isotropic Hardening Model

The change in the size of the yield surface is modelled using an exponential law [42] as below:

Exponential Law:

(5) $$\sigma^{0}=\sigma {¦}_{0}+Q_{\infty }\left(1-e^{-b{\bar{\varepsilon }}^{pl}}\right)$$

where $\sigma {¦}_{0}$ is the yield at zero plastic strain, $Q_{\infty }$ is the maximum change in the size of the yield surface and b is the rate at which the size of the yield surface changes as plastic straining develops.

$C_{k},\gamma_{k},\;\sigma {¦}_{0},Q_{\infty },\;\;b$ are all the material parameters that are calibrated against cyclic test data. The temperature dependent model parameters are determined by minimizing the root mean square of the error between the experimental data and the simulated response using the Nedler–Mead optimization algorithm. 

The cost function for minimizations is as shown below:

(6) $$F\;(\pi )\;=\;\frac{1}{2}\;\sum_{i=1}^{N}{\left[\sigma_{i}\left(\pi ,t_{i}\right)-\bar{\sigma }\left(t_{i}\right)\right]}^{2}$$

With σ_i_ being the simulated value and $\bar{\sigma }$, the experimental response. The data are sampled at different time intervals t_i_ equating to identical strain load levels in the simulated and experimental response. The model parameters are calibrated against test data of the first 20 cycles of strain controlled LCF tests with ε_amp_ = 0.4% and a strain ratio of R_ε_ = −1 at the three different tested temperatures of 150, 200 and 250 °C. The model is calibrated against one set of experimental data at each temperature. The summary of the obtained model parameters is presented in Table 5.

**Figure 27:**
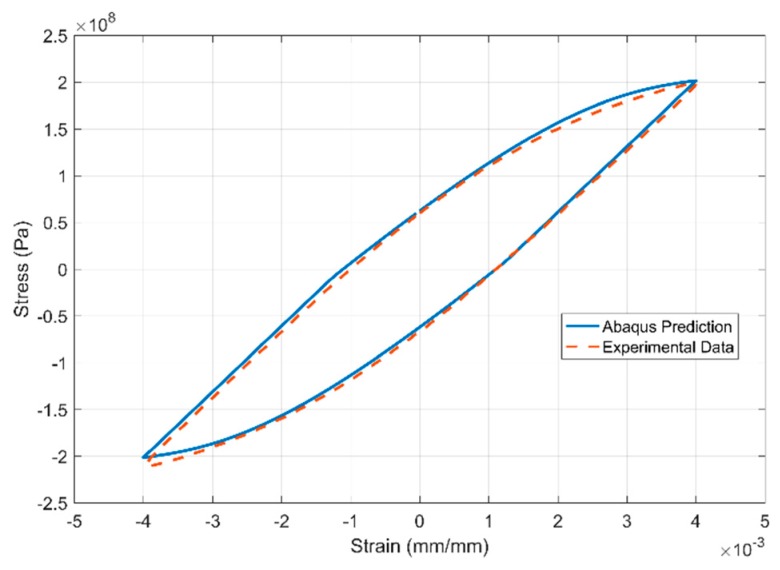
Model prediction vs. experimental data at 150 °C of the 20^th^ strain cycle.

**Figure 28:**
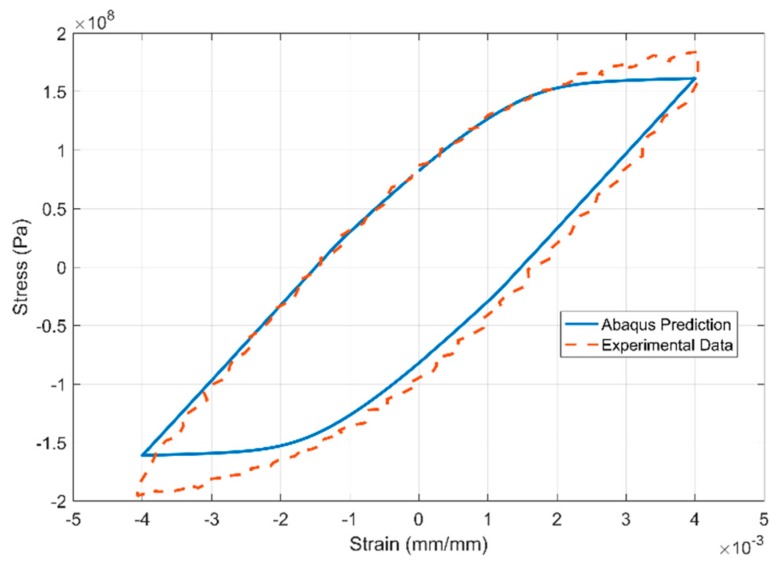
Model prediction vs. experimental data at 200 °C of the 20^th^ strain cycle.

**Figure 29:**
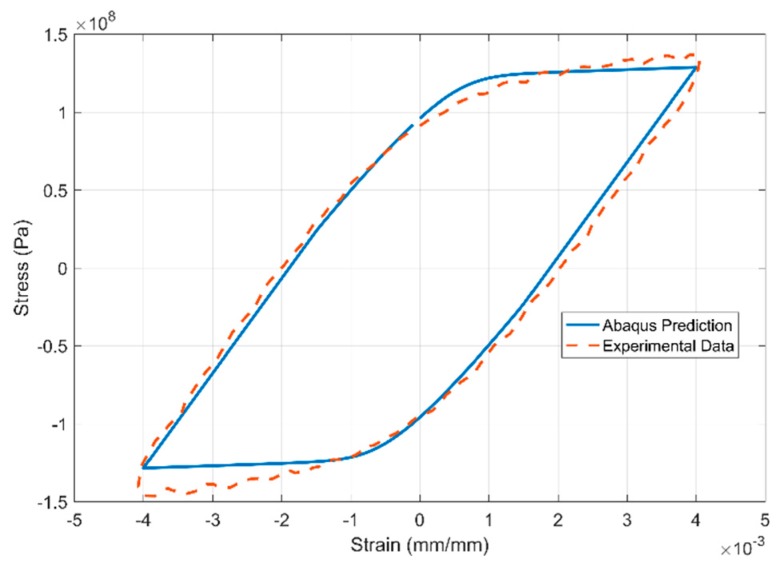
Model prediction vs. experimental data at 250 °C of the 20^th^ strain cycle.

The results of the inverse parameter identification showing the 20th cycle of the experimental data against the simulation results obtained from commercial FE software Abaqus are presented in Figure 27, Figure 28 and Figure 29 for the strain-controlled tests at 150, 200 and 250 °C respectively. The temperature dependent model parameters are obtained by calibrating the model against the test data from 150 °C and using the obtained values as the starting values in the optimization procedure for 200 °C and so on. The optimization routine is constrained to obtain sequentially varying values to get predictable interpolation at temperatures in between the tested and modelled values. Such constraining is often necessary to avoid obtaining local minima that would otherwise lead to unpredictable interpolations.

#### 4.3.3. Fatigue Life Criterion

The load–response and the corresponding damage to the material structure could either be coupled or uncoupled. Coupled approaches, while seemingly attractive from a computational point of view are difficult to calibrate on account of the large number of parameters and also, do not necessarily provide improved predictions [17,43]. The failure criteria in a thermo-mechanical context should be capable of predicting the failure in applications with transient operating temperatures and isothermal loads under uniaxial and multi-axial load conditions [1]. In an industrial context, a decoupled approach with classical failure criteria like the Coffin–Manson relation or more recent applications of energy criteria like plastic dissipated energy, etc., are often used to predict the life of the components [1,15]. The life of the A356–T7 aluminium alloy cylinder heads is found to be often dictated by the casting defects and other microstructural features like the secondary dendrite arm spacing, the morphology and size of the secondary particles [15]. Studies on fracture surfaces by Fuoco et al. [8] on fatigue cracks in aluminium cylinder heads show cracks originating from micro-porosities and oxide film inclusions. Fatigue damage studies by Koutiri et al. [23,44] on A356–T7 alloys identified two competing fatigue damage mechanisms with the micro-shrinkage pores playing a very fundamental role in dictating the fatigue behaviour. In the absence of the micro-shrinkage pores, the fatigue behaviour was controlled by other microstructural inhomogeneities like the secondary precipitates that affect the crack initiation. In addition, they found no significant difference in the fatigue strength of the material under uniaxial and biaxial stress states in A356 + 0.5% Cu–T7 group of alloys contrary to the prediction of most multi-axial fatigue criteria [23,44].

##### Coffin–Manson Relation

On account of the local yielding and plasticity often involved in thermo-mechanical fatigue (TMF), a strain-based approach to fatigue life prediction is frequently adopted in the automotive industry considering the cyclical nature of the thermal stresses associated with the engine start-stop cycle and the ductile nature of the aluminium alloys used in the internal combustion (IC) engines. A temperature dependent strain life model is developed and can be employed to predict the TMF life of components.

**Figure 30:**
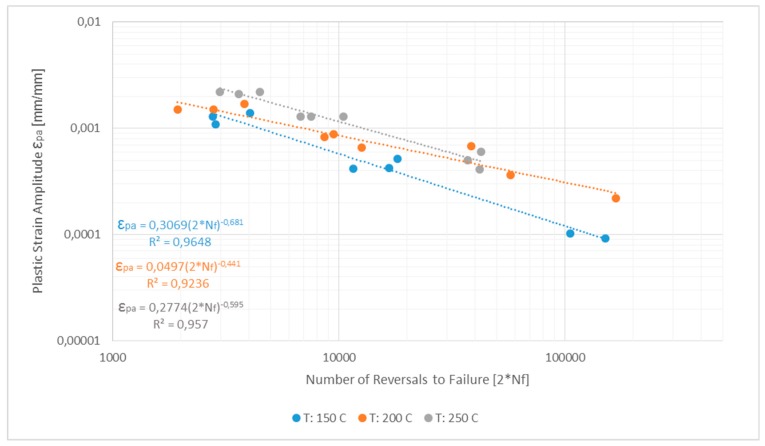
Plot of the plastic strain amplitude vs. the load reversals (2*N_f_) to failure at elevated strain controlled LCF tests.

The Coffin–Manson plot determined from completely reversed strain-controlled fatigue tests, R_ε_ = −1 at different temperatures is plotted in Figure 30. The strain life model data are presented in Table 6 with the strain life model being an additive partition of the elastic and plastic strain components, i.e., ε_a_ = ε_ea_ + ε_pa_. The elastic component (ε_ea_) is determined from the stress amplitude and by using the Hooke’s law while the plastic strain amplitude (ε_pa_) is taken to be half the width of the half-life hysteresis loops.

##### Coffin–Manson Relation

(7) $$\varepsilon_{a}\;=\;\frac{\sigma_{f}^{\prime }}{E}\;{\left(2N_{f}\right)}^{b}+{\;\varepsilon }_{f}^{\prime }\;{\left(2\mathrm{Nf}\right)}^{c}$$

With ε_ea_ = $\frac{\sigma_{a}}{E}$ = $\frac{\sigma_{f}^{\prime }}{E}\;{\left(2N_{f}\right)}^{b}$ and ε_pa_ = $\varepsilon_{f}^{\prime }\;{\left(2\mathrm{Nf}\right)}^{c}$.

The obtained test data are modelled against the above relation and the model parameters as determined from the hysteresis loops at half the life (N_f_/2) of the tested samples are presented in Table 6. Since the tests have been carried with low loads to obtain long lives, the obtained data can be used to predict lives where little plasticity is involved and long lives are expected to form a wide-ranging approach to fatigue life prediction. The two different life curves often dominate depending on the loading situation with the life curve aligning along the elastic strain-life line at long lives and along the plastic strain-life line at short lives. 

With increase in temperature, the ε_f_′ increases and the σ_f_′ decreases indicating an increasingly ductile behaviour with increasing temperatures also reinforced by the corresponding increase in transition fatigue life. 

Azadi et al. [35] studied the low cycle fatigue properties of the peak aged A356 - T6 alloy with significantly lower copper addition (0.01%) and on comparison, the fatigue lives reported at 250 °C are significantly lower at comparable strain levels in relation to the overaged A356 + 0.5% Cu–T7 alloy studied here. The fatigue lives at lower temperature of 200 °C are comparable however indicating the potentially improved fatigue lives of the A356 alloy with higher copper content at elevated temperatures similar to improved monotonic deformation properties at temperatures above 250 °C as reported in various other studies [6,7].

The presented deformation and fatigue life models can be used for estimating the load-response history and the structural durability of components made with the A356–T7 alloy within the temperature ranges presented. The fatigue life curves developed span a range of tests with both significant and very low plasticity making it suitable for both low and high cycle fatigue life evaluations using CAE procedures.

## 5. Conclusions

A356–T7 + 0.5% Cu alloy samples extracted from the valve bridge areas of the cylinder heads of internal combustion engines were subjected to uniaxial monotonic and cyclic strain-controlled tests to study their deformation and fracture properties. Dilatometric test was carried out to study the thermal expansion behaviour of the alloy.

- The material exhibits decreasing strength and increasing ductility with increasing temperatures under monotonic loading. The material exhibits strain hardening at temperatures at and below 150 °C and a strain softening at temperatures above 150 °C under uniaxial tensile loading.
- The material exhibits cyclic softening with strain load cycles at all the tested temperatures of 150, 200 and 250 °C. The tests at elevated temperatures show reduced stress response and following increased plastic strains amplitudes.
- Dilatometry reveals a fairly constant coefficient of thermal expansion measured varying between (25–26) × 10^−6^ °C^−1^ in the temperature range 25–250 °C.
- The monotonic and cyclic stress–strain curves exhibiting no significant yield point can be modelled accurately with a Ramberg–Osgood type model
- The cyclic deformation behaviour can be modelled using a temperature dependent non-linear combined kinematic and isotropic model with one linear and one non-linear backstress.
- The scatter in mechanical properties measured is influenced by the test temperature with the difference between replicas decreasing with increasing temperatures.

## Figures and Tables

**Table 1:** Chemical composition of A356 + 0.5% Cu–T7 cast aluminium alloy as tested in wt.%.

Si	Cu	Mg	Ti	Fe	Mn	B	Others	Al
6.8	0.53	0.35	0.12	0.10	0.07	0.0012	<0.05	Bal

**Table 2:** Summary of the mechanical and thermal tests.

Series	Nature of Test	Loading Conditions	Number of Tests/Replicas
1	Room Temperature Tensile Tests	Temperature: Room temperature (RT) Strain Rate: 0.01% s^−1^	3
2	High Temperature Tensile Tests	Temperatures: 100, 150, 200, 250, 300 °C Strain Rate: 0.01% s^−1^	2
3	Uniaxial, completely reversed, total strain-controlled fatigue tests	Temperatures: 150, 200, 250 °C Strain Rate: 1% s^−1^	2-3
4	Dilatometry	Temperature Range: 25–360 °C Heating Rate: 1 °C min^−1^ Atmosphere: Nitrogen Contact Pressure: 30 cN	1

**Table 3:** Summary of tensile properties of A356 + 0.5% Cu–T7 (mean values based off true stress and true strain values of the tested replicas).

Temperature °C	Young’s Modulus *E* [GPa]	Offset Yield Strength *R_p_* 0.2% [MPa]	Ultimate Tensile Strength *R_m_* [MPa]	Maximum Strain before Fracture *ε_f_* [%]
RT	72	211	273	4.7
100	71	199	247	7.5
150	70	184	217	13.4
200	64	158	180	15.2
250	61	122	133	17.2
300	56	73	84	>17.2

**Table 4:** Ramberg–Osgood model parameters for cyclic hardening of A356 + 0.5% Cu–T7.

Temperature	Young’s Modulus [GPa]	Offset Yield Strength $\sigma_{0}^{\prime }$ [MPa]	*H′* [MPa]	Cyclic Strain Hardening Coefficient *n′*
150 °C	70	192	274	0.0572
200 °C	64	177	321	0.0955
250 °C	61	124	216	0.0889

**Table 5:** Model parameters of the non-linear combined isotropic–kinematic hardening model.

Temp. °C	Young’s Modulus [Pa]	Yield Stress at Zero Plastic Strain and Equiv. Stress (For Isotropic Hardening Model) [Pa]	Kinematic Hardening Parameter C1 [Pa]	Gamma 1 [–]	Kinematic Hardening Parameter C2 [Pa]	Gamma 2 [–]	Q-Infinity [Pa]	Hardening Parameter b [–]
150	70.22 × 10^9^	9.967 × 10^7^	1.786 × 10^11^	1651	1.551 × 10^9^	0	−20 × 10^6^	2.0
200	64.13 × 10^9^	9.5 × 10^7^	1.997 × 10^11^	2933	1.569 × 10^9^	0	−20 × 10^6^	2.2
250	60.82 × 10^9^	8.2 × 10^7^	2.034 × 10^11^	4155	1.581 × 10^9^	0	−20 × 10^6^	2.3

**Table 6:** Coffin–Manson model parameters of A356 + 0.5% Cu–T7 alloy at various temperatures.

Temperature [°C]	E [GPa]	σ_f_′ [MPa]	b	ε_f_′	c	Transition Fatigue Life N_t_
150	70	421	−0.096	0.3069	−0.681	2727
200	64	346	−0.087	0.0497	−0.441	3745
250	61	231	−0.073	0.2774	−0.595	14004
